# Genetic differentiation and asymmetric gene flow among Carpathian brown bear (*Ursus arctos*) populations—Implications for conservation of transboundary populations

**DOI:** 10.1002/ece3.4872

**Published:** 2019-01-23

**Authors:** Maciej Matosiuk, Wojciech Śmietana, Magdalena Czajkowska, Ladislav Paule, Jozef Štofik, Diana Krajmerová, Andriy‐Taras Bashta, Stefan Jakimiuk, Mirosław Ratkiewicz

**Affiliations:** ^1^ Institute of Biology University of Bialystok Białystok Poland; ^2^ Institute of Nature Conservation PAS Kraków Poland; ^3^ Faculty of Forestry Technical University Zvolen Slovakia; ^4^ National Park Poloniny Stakčín Slovakia; ^5^ Institute of Ecology of the Carpathians National Academy of Sciences of Ukraine Lviv Ukraine; ^6^ WWF Poland Warszawa Poland; ^7^ Present address: KRAMEKO Kraków Poland

**Keywords:** brown bear, Carpathians, conservation, genetic structure, phylogeography, transboundary populations

## Abstract

The abundance and distribution of large carnivores in Europe have been historically reduced. Their recovery requires multilevel coordination, especially regarding transboundary populations. Here, we apply nuclear and mitochondrial genetic markers to test for admixture level and its impact on population genetic structure of contemporary brown bears (*Ursus arctos*) from the Eastern, Southern, and Western Carpathians. Carpathian Mountains (Europe). Nearly 400 noninvasive brown bear DNA samples from the Western (Poland) and Eastern Carpathians (Bieszczady Mountains in Poland, Slovakia, Ukraine) were collected. Together with DNA isolates from Slovakia and Romania, they were analyzed using the set of eight microsatellite loci and two mtDNA regions (control region and cytochrome *b*). A set of 113 individuals with complete genotypes was used to investigate genetic differentiation across national boundaries, genetic structuring within and between populations, and movement between populations. Transboundary brown bear subpopulations (Slovakia and Poland) did not show significant internal genetic structure, and thus were treated as cohesive units. All brown bears from the Western Carpathians carried mitochondrial haplotypes from the Eastern lineage, while the Western lineage prevailed in the brown bears from the Bieszczady Mountains. Despite similar levels of microsatellite variability, we documented significant differentiation among the studied populations for nuclear markers and mtDNA. We also detected male‐biased and asymmetrical movement into the Bieszczady Mountains population from the Western Carpathians. Our findings suggest initial colonization of the Western Carpathians by brown bears possessing mtDNA from the Eastern lineage. Genetic structuring among populations at microsatellite loci could be a result of human‐mediated alterations. Detected asymmetric gene flow suggests ongoing expansion from more abundant populations into the Bieszczady Mountains and thus supports a metapopulation model. The knowledge concerning this complex pattern can be implemented in a joint Carpathian brown bear management plan that should allow population mixing by dispersing males.

## 1 INTRODUCTION

The brown bear (*Ursus arctos* L.) is the largest terrestrial carnivore with a wide Holarctic distribution. Its populations formerly occupied all of Europe (Swenson, Gerstl, Dahle, & Zedrosser, 2000; Swenson, Taberlet, & Bellemain, 2011). However, since the 19th century, habitat destruction and human persecution have led to a severe decline among European brown bear populations, resulting in local extirpations and population fragmentation (Servheen, 1990). In western and central Europe, few contemporary populations are isolated from each other and from the western limit of continuous brown bear range located in Scandinavia, Estonia, and western Russia (Zedrosser, Dahle, Swenson, & Gerstl, 2001). The Carpathian population of brown bears spans national boundaries of several countries, consequently, its conservation and management falls under different jurisdictions. Therefore, the challenge of maintaining this wide‐ranging carnivore is heightened (Chapron et al., 2014; Swenson et al., 2000, 2011). The importance of this population is even greater because the Carpathians could have served as a refuge area or as a crucial movement corridor for brown bears, which led to the rise of brown bear populations in eastern and northern Europe during or after the last ice age (Saarma et al., 2007).

Mitochondrial DNA (mtDNA) diversity for brown bear has been extensively researched to elucidate phylogeographic trends in Europe (Anijalg et al., 2018; Davison et al., 2011; Hewitt, 2004; Keis et al., 2013; Korsten et al., 2009; Leonard, Wayne, & Cooper, 2000; Miller, Waits, & Joyce, 2006; Taberlet, Fumagalli, Wust‐Saucy, & Cosson, 1998). A current phylogeographical study, (Anijalg et al., 2018) based on over 250 complete mitochondrial genomes, identified seven mtDNA clades, each with several subclades. Clade 3a1 (Eastern lineage) was found to be the most widely distributed from Scandinavia, throughout northern continental Asia, to Alaska. The study estimated that this subclade originated approximately 45 kya ago and diversified in Europe about 25 kya. Interestingly, Valdiosera et al. (2008) showed the presence of Eastern lineage haplotypes in Iberia during the late Pleistocene, 80 kya. Thus, one could expect a more westward distribution of the Eastern lineage, especially in the Western Carpathians. Indeed, earlier and recent phylogenetic studies revealed the presence of Eastern lineage (clade 3a1) in contemporary brown bear populations from Slovakia (Anijalg et al., 2018; Benazzo et al., 2017; Paunović & Ćirović, 2006; Taberlet & Bouvet, 1994). However, Benazzo et al. (2017) showed that the nuclear genomes of brown bears from Slovakia exhibit genetic affinity with Apennine, Alpine, Iberian, Balkan, and Scandinavian bears.

**Figure 1:**
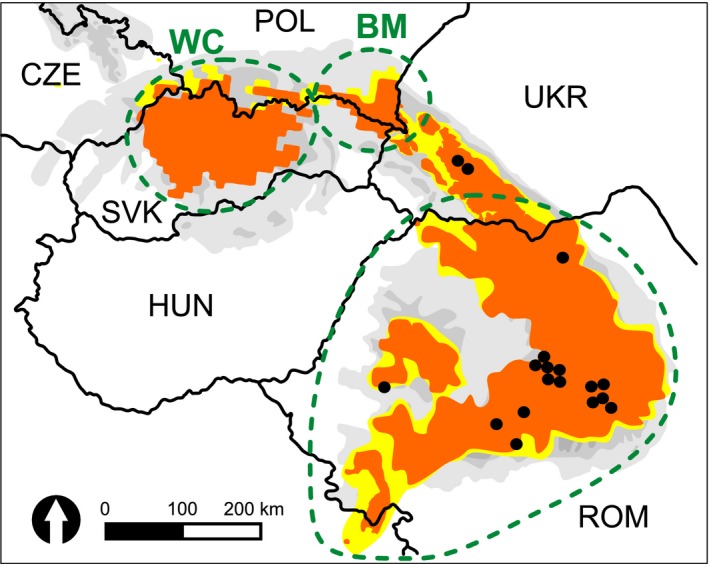
Distribution of brown bear in Carpathians (based on Dykyy & Shkvyria, 2015; Kaczensky et al., 2012; A.‐T. Bashta unpublished materials—brown bear observations in Ukraine during last 10 years) and populations studied: Western Carpathians (WC), Bieszczady Mountains (BM), and Romanian Carpathians (ROM). Permanent presence is indicated with orange, while sporadic occurrence with yellow. Individuals sampled in Romania and Ukraine are indicated with black dots

In addition to natural processes, human‐mediated alterations resulted in severe brown bear population bottlenecks (Taberlet & Bouvet, 1994). Local persecutions, significant habitat fragmentation, followed by intentional translocations could have a disproportional role in shaping genetic diversity of this wide‐ranging carnivore (Bray et al., 2013; Crispo, Moore, Lee‐Yaw, Gray, & Haller, 2011). Carpathian Mountains population is the largest brown bear population in Europe outside the continuous distribution range (Figure 1a; Chapron et al., 2014). However, in the early 20th century, it experienced a severe decline due to extensive deforestation and overhunting which led to the isolation of a small population of brown bears in Western Carpathians from the rest of the population (Finďo, Skuban, & Koreň, 2007; Hartl & Hell, 1994). At the end of World War I, the Western Carpathian population of brown bears decreased to roughly 15–75 individuals (after Straka, Paule, Ionescu, Štofík, & Adamec, 2012). In contrast, the East‐Carpathian population, resident in Romania, Ukraine, South‐Eastern Poland, and North‐Eastern Slovakia, never dropped below 800 individuals (Ionescu, 1999). However, its westernmost portion (Poland and Slovakia) was limited to only several individuals between World War I and World War II (Jakubiec, 2001; Sabadoš & Šimiak, 1981). Changes in management policies and protective legislation have allowed for the restoration of the Carpathian brown bear population (Chapron et al., 2014). In the West‐Carpathian population, recent estimates indicate that the population is stable (approx. 1,250 individuals; Kaczensky et al., 2012; Paule et al., 2015), with a majority of bears in Slovakia and approximately a dozen or so in Poland. The East‐Carpathian population in the Bieszczady Mountains is much smaller with approximately 83 individuals in Poland (Śmietana et al., 2014) and at least 15 individuals in Slovakia (Straka, Štofík, & Paule, 2013). The Ukrainian part of the East‐Carpathian population is occupied by at least 400 brown bears (Zedrosser et al., 2001). The largest population of the Carpathians, which includes portions of the Eastern and Southern Carpathians, harbors more than 6,000 brown bears in Romania (Chapron et al., 2014; Kaczensky et al., 2012).

Population genetic studies using nuclear microsatellite markers revealed the presence of strong genetic structuring between populations from Western Slovakia, Eastern Slovakia, and Romanian Carpathians (Straka et al., 2012). The authors concluded that the present genetic differentiation is a result of nearly 100‐year isolation of these geographically close populations; however, they did not estimate levels of gene flow among these populations. In this study, mitochondrial and autosomal markers were used to measure genetic variability within and differentiation among brown bear populations from Western Carpathians (WC; Poland and Slovakia), Bieszczady Mountains (BM; Poland and Slovakia), Ukraine (UKR; mtDNA only), and Romania (ROM). We also estimated the level and direction of admixture that might be indicative for both natural male‐biased dispersal and translocations. Moreover, we tested for a possible asymmetry in gene flow, as it could be an indicator of a metapopulation model of genetic connectivity during the recolonization process. Finally, since brown bear populations in Poland (POL) and Slovakia (SVK) are transboundary populations that experience different management practices (the brown bear has been strictly protected in Poland since 1952, while in Slovakia the species is managed by culling), we compared subsamples that originated from these two countries for conservation purposes.

## 2 MATERIAL AND METHODS

### 2.1 Samples

In our analysis, DNA was extracted from the following sources: scats (*n = *244); hairs found on marking trees and fences crossed by bears (*n* = 134); dried blood found on a vehicle destroyed by a bear; 14 buccal swabs or hairs collected from bears captured for telemetry studies; an individual rescued from poacher's snare; another one captured due to public safety; and from four dead individuals occasionally found in the field. Samples were collected in Polish (*n* = 376), Slovakian (*n* = 20), and Ukrainian (*n* = 3) Carpathians. Preliminary results of microsatellite DNA analyses of a subsample from Poland were reported by Śmietana, Rutkowski, Ratkiewicz, and Buś‐Kicman (2012). We also used DNA isolates obtained from brown bears sampled in Slovakia (*n = *35) and Romania (*n* = 16) that were previously analyzed by Straka et al. (2012). In total, we used 452 samples: 44 from Western Carpathians (POL and SVK), 387 from the Bieszczady Mountains (POL and SVK), 16 from Romanian Carpathians, three samples from Ukrainian Carpathians, and two samples from Greece (Figure 1). The last two samples were used in a mtDNA survey and their haplotypes were used in network analysis only.

### 2.2 Laboratory procedures

Two classes of genetic markers were used: nuclear microsatellite DNA and mitochondrial DNA (mtDNA). Microsatellite DNA analyses of the first set of samples (*n* = 331) were performed in the Laboratory of Molecular Biology (Institute of Biology, University of Białystok), and the second set of samples (*n* = 121, all from Eastern Carpathians) were performed by Wildlife Genetics International (Nelson, Canada). Within analyses of the first set of samples, we chose and organized 12 autosomal and unlinked microsatellite markers (Bellemain & Taberlet, 2004; Paetkau, Calvert, Stirling, & Strobeck, 1995; Straka et al., 2012; Taberlet et al., 1997) into three multiplex sets that maximize the number of loci suitable for simultaneous amplification. Set 1 consisted of 5 loci: G10M, G10J, Mu09, Mu61, and Cxx20; Set 2 consisted of 4 loci: G10B, G10C, Mu51, and Mu59; Set 3 amplified 3 loci: Mu10, Mu11, and G10X. In addition, we amplified a ~76 bp fragment of the *Sry *gene of males (Straka et al., 2012; Taberlet et al., 1997) together with microsatellites in Set 2 in order to genetically identify the sex of the individuals studied. Multiplex PCRs were performed with GeneAmp PCR System 9,700 (Applied Biosystems) in 10 μl reaction volume containing 2 μl of isolated genomic DNA, 4.5 μl Qiagen Multiplex PCR Master Mix (1×), 0.9 μl mix of primers (0.2 μM of each primer), and 2.6 μl RNase‐free water. Each multiplex PCR started with an initial activation step at 95°C for 15 min, followed by 42 cycles, with denaturation at 94°C for 30 s, annealing for 90 s (at 49.6°C for Set 1, 58°C for Set 2, and 54°C for Set 3), extension at 72°C for 60 s, and final extension at 60°C for 30 min. The PCR products were mixed with 10 μl ultragrade formamide and 0.2 μl GeneScan 500‐LIZ size standard (Applied Biosystems), denatured at 95°C for 5 min, rapidly cooled and detected using ABI 3,130 Genetic Analyzer (Applied Biosystems). The allele fragment lengths were estimated using the Auto Bin feature in genemapper 4.0 software (Applied Biosystems). Four microsatellite loci (Cxx20, Mu10, Mu51, and G10X) produced unreliable results for stool samples (no amplification, irregular stuttering, or artefacts affecting scoring) and therefore were discarded from multiplex sets after initial screening. PCR reactions were performed from two to four times depending on the consistency of obtained PCR products. During initial screening, samples with <4 genotyped loci were removed and not included in any further analysis. For samples which passed this step but did not produce reliable genotypes at one up to four loci, we attempted to fill in missing or weak data by performing additional PCRs (in multiplex sets or targeting single loci). All pairs of remaining unique genotypes were compared in search of similar genotypes showing one to maximum of three mismatches, then scrutinized for potential scoring errors. If necessary, samples were rerun in selected markers. Each group of matching multilocus genotypes was considered an individual animal. The frequency of null alleles was assessed in cervus 3.0.3 (Kalinowski, Taper, & Marshall, 2007), and allelic dropout rates were estimated using Gimlet 1.3.3 (Valière, 2002). For the remaining eight microsatellite loci, the frequencies of null alleles were low to moderate (Supporting Information Table S1) and thus these loci were used for further analyses. The allelic dropout rate was estimated at 0.049 across loci and depended on allele size of the particular loci (Supporting Information Table S1).

The second set of samples was analyzed by Wildlife Genetics International (WGI) with the use of the same set of eight selected microsatellite markers, and ZFX/ZFY marker for sex identification (instead of *Sry* used in Laboratory of Molecular Biology). All microsatellite loci analyzed by WGI were amplified separately. Genotype scores were obtained following protocols for low DNA quality and quantity samples, highly relying on: (a) contamination prevention, (b) strict sample quality control and scoring convention, (c) error‐checking and considering samples with missing data on a case‐by‐case basis, and (d) extensive comparisons of similar genotypes followed by reamplification and reanalysis if necessary (for detailed information see Paetkau, 2003). To allow coalescence of data, 18 good quality hair samples representing the same individuals were genotyped by the two laboratories. Obtained genotype scores for these samples were compared and conversion factors for each marker were identified. It allowed for the conversion of all genotype scores into one database. The consistency of identification of males and females using the *Sry* and ZFX/ZFY markers were checked between the laboratories. In total, complete genotypes of 33 individuals were obtained from at least two independent samples (range: 2–45), while remaining individuals were identified based on one sample.

MtDNA analyses were only performed in the Laboratory of Molecular Biology. The partial sequence of the cytochrome *b* (345 bp) gene was amplified together with flanking tRNA‐Thr (70 bp) and tRNA‐Pro (9 bp) using newly designed primers in fastpcr (Kalendar, Lee, & Schulman, 2009) (cyt*b*_F—CCGACTTACTAGGAGACCCTGA, tRNA‐Pro_R—TAGTGGAGCTGTTGCTTCTTCCT). In addition, the amplification of 385–388 bp of the mitochondrial control region together with 10 bp of tRNA‐Pro were performed using primers CR_F—AGGAAGAAGCAACAGCTCCACTA, CR_R—CCATCGAGATGTCCCATTTGAAG. The PCRs for both fragments were performed in 5 µl reaction volume containing 2 µl genomic DNA (~20 ng), 1.7 µl Qiagen Multiplex PCR Master Mix (1×), 0.3 µl mix of primers (0.2 µM of each primer), and 1 µl RNase‐free water. The reaction conditions followed the same protocol as used for the microsatellites, it ran for 40 cycles with annealing at 57°C. Sequencing reactions in both directions were performed using the BigDye™ Terminator Cycle Sequencing Kit (Applied Biosystems). The reaction conditions were as follows: 25 cycles with denaturation at 95°C for 20 s, annealing at 50°C for 15 s, extension at 76°C for 60 s. The detection of sequencing reaction products was carried out on ABI 3,130 Genetic Analyzer. Sequences were aligned manually in the bioedit sequence editing program (Hall, 1999). Sequences of all haplotypes have been submitted to the GenBank databases under accession numbers: MG254039–MG254048 (for mtDNA cytochrome *b* gene) and MG254049–MG254058 (for mtDNA control region).

### 2.3 Phylogenetic analyses

To test phylogenetic relationships among concatenated cytochrome *b* and control region haplotypes (809–813 bp long), we constructed a neighbor‐joining tree with mega v.5.05 (Tamura et al., 2011) with 1,000 bootstrap replicates used to assess support for tree nodes. All indels found within mtDNA control region sequences were excluded from phylogenetic and population genetics analyses. We also used brown bear cyt *b* and mtDNA control region sequences deposited in GenBank (from Bon et al., 2008; Keis et al., 2013; Miller et al., 2012; Taberlet & Bouvet, 1994). *Ursus americanus* (AF303109) was used as an outgroup. Haplotype network reconstruction was performed in network v4.6.1.0 (Bandelt, Forster, & Röhl, 1999). We also calculated net pairwise divergence (Da) among mtDNA lineages using mega v.5.05.

### 2.4 Population genetics

We used cervus 3.0.3 (Kalinowski et al., 2007) and fstat 2.9.3 (Goudet, 1995) to estimate the number (*A*) and average number of alleles per locus (*N*_A_), allelic richness (*A*_R_), allele size range, gene diversity (*G*_D_), departures from Hardy–Weinberg equilibrium (HWE), and inbreeding coefficient (*F*_IS_) in brown bear populations studied. Linkage disequilibria (LD) between pairs of loci were estimated and corresponding tests were based on permutations in fstat 2.9.3.

We further applied a Bayesian clustering approach to infer the number of populations using the software structure 2.2.3 (Pritchard, Stephens, & Donnelly, 2000) without prior information of the sampling locations. We assumed the admixture model with correlated allele frequencies, and specified burn‐in of 1,000,000 iterations and 5,000,000 Markov Chain Monte Carlo (MCMC) replicates. The program was run 10 times for each *K*, between 1 and 5. structure was also used to estimate the most probable number of genetically distinct populations (*K*), admixture level between them and to detect possible migrants. The test for migrants was additionally computed with geneclass2 (Piry et al., 2004) based on the algorithm of Paetkau et al. (1995). We also estimated the migration rate (*m*) using the Bayesian assignment algorithm implemented in BayesAss (Wilson & Rannala, 2003) to consider short‐term gene flow. The MCMC method was run for 20,000,000 iterations with a recommended burn‐in period of 1,000,000 and a sampling frequency of 2,000 iterations. The run used was adjusted based on preliminary runs, identical delta value (i.e., maximum parameter change per iteration) of 0.10 for allele frequency, migration, and inbreeding. Mitochondrial DNA haplotypes of the identified F0 immigrants were checked for consistency of mtDNA and microsatellite data. The spatial distribution of immigrants was analyzed, as well as their relatedness in kingroup, (Konovalov, Manning, & Henshaw, 2004) in order to reveal possible geographic patterns of dispersal and check if dispersal events were random.

For mtDNA analysis, we calculated the number of haplotypes (*Nh*), haplotype diversity (*h*), nucleotide diversity (*π*), number of segregating sites (*S*), and mean number of pairwise differences (PD) for concatenated mtDNA control region and cyt *b* using software packages arlequin (Excoffier & Lischer, 2010) and dnasp v.5 (Librado & Rozas, 2009). Genetic differentiation between the studied brown bear populations was assessed by pairwise *F*_ST_ and *φ*_ST_ values based on pairwise differences for mtDNA, while average *F*_ST_ and *R*_ST_ values were used for microsatellite loci. All values were statistically tested in arlequin.

## 3 RESULTS

### 3.1 Genetic polymorphism and admixture at nuclear loci

Out of the 275 samples of brown bears from Poland analyzed in the Laboratory of Molecular Biology, we obtained reliable genotypes for 129 samples (49%) that corresponded to 56 individuals. While 121 samples analyzed by Wildlife Genetics International yielded 94 reliable genotypes (78%) which corresponded to 41 individuals. Nineteen out of these 41 individuals were identified earlier by the Laboratory of Molecular Biology (they were present in the first set of samples). Thus, with an addition of DNA isolates from Slovakia and Romania, our microsatellite analyses were based on 121 individuals while mtDNA analyses augmented by the samples from Ukraine comprised of 119 individuals (for detailed information about sample usage see Supporting Information Table S2). There was a full consistency of sex identification using *Sry* and ZFX/ZFY markers for the samples studied in both labs.

For the total sample, the number of alleles per locus (*A*) ranged from 6 (at 3 loci) to 13 alleles (Mu59); on average 8.63 alleles (Supporting Information Table S1). For the eight microsatellite loci studied, the allele numbers and their size ranges were very similar in three studied brown bear populations (Supporting Information Table S1), implying genetic affinity among them. The gene diversity at the studied loci ranged from 0.722 to 0.795 and *F*_IS_ values in brown bear populations were not significantly different from zero (Table 1). The majority of tests for LD and all HWE tests did not identify statistically significant evidence of departures from the expectations of random mating. The only significant LD were found in the Bieszczady Mountains population for three pairs of loci: Mu61 and G10M, Mu61 and G10B, G10B and Mu11. The average number of alleles per locus was the lowest in the sample from Polish Western Carpathians and the highest in the sample from Romanian Carpathians (*N*_A_ = 4.25 and 7.13, respectively, Table 1). The genetic variability values are given in Table 1 and they did not differ significantly (1,000 permutations in fstat at *p* > 0.05) among the studied brown bear populations.

**Table 1:** Measures of genetic variation for eight microsatellite loci in the studied brown bear from Western Carpathians (WC), Bieszczady Mountains (BM), and Romanian Carpathians (ROM)

Population	*n*	*N*_A_	*A*_R_	*G_D_*	*F*_IS_	HWE
WC						
POL	7	4.25	NE (3.93)	0.746	0.083	NE
SVK	16	5.75	5.59 (4.15)	0.734	–0.019	NE
POL + SVK	23	5.88	5.42 (NE)	0.738	0.008	ns
BM						
POL	66	7.00	5.51 (4.16)	0.719	–0.008	NE
SVK	17	5.13	5.13 (4.04 )	0.736	–0.043	NE
POL + SVK	83	7.00	5.49 (NE)	0.722	–0.013	ns
ROM	15	7.13	7.06 (4.87)	0.795	0.095	ns
Total	121	8.63	6.38 (NE)	0.769	0.050	NE

Note

*n*: sample size; *N*_A_: average number of alleles per locus; *A*_R_: allelic richness based on minimum sample size of 14 individuals (values in parentheses are based on minimum sample size of five individuals); *G*_D_: gene diversity; *F*_IS_: inbreeding coefficient (all values not different from zero at *p* > 0.05); HWE: Hardy–Weinberg equilibrium; ns: not significant at any loci, Bonferroni corrected and in global test; NE: not evaluated.

We did not find significant genetic differentiation at microsatellite loci between the “transboundary subsamples” in Western Carpathians in Poland and Slovakia (*F*_ST_ = 0.010, *p* > 0.05) as well as between Polish and Slovakian parts of Bieszczady Mountains (*F*_ST_ = 0.005, *p* > 0.05). Thus, the studied samples from Poland and Slovakia were pooled and regarded as two genetically distinct groups: Western Carpathians (POL and SVK, *n* = 23) and Bieszczady Mountains (POL and SVK, *n* = 83, ca. 80% of census size) for further analyses. Pairwise genetic differentiation values for microsatellite loci between brown bear populations from the Western Carpathians and Bieszczady Mountains were moderate for *F*_ST_ (0.106) or great (*R*_ST_ = 0.197) and significantly different from zero at *p* < 0.001. The genetic differentiation values between the population from Romania and the other two populations were low to moderate (Table 2) and significantly different from zero. The average *F*_ST_ among the studied three brown bear populations was moderate (0.092, 95% CI: 0.069–0.116, *p* < 0.001) while *R*_ST_ was great (0.162, *p* < 0.001).

**Table 2:** Genetic differentiation in eight microsatellite loci: *F*_ST_ (above diagonal) and *R*_ST_ (below diagonal) between brown bear populations from Western Carpathians (WC), Bieszczady Mountains (BM), and Romanian Carpathians (ROM)

*F*_ST_/*R*_ST_	WC	BM	ROM
WC	–	0.106	0.039
BM	0.197	–	0.085
ROM	0.017	0.140	–

**Figure 2:**
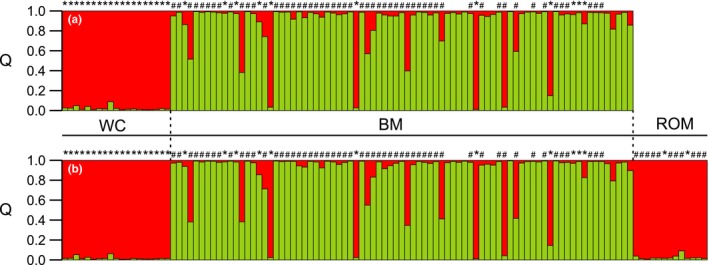
Genetic structuring of *Ursus arctos* populations from Western Carpathians (WC), Bieszczady Mountains (BM), and Romania (ROM) inferred by structure for *K* = 2 groups. Individual assignment to each of two genetic clusters (shown in red and green) was assessed: with the use of samples from Western Carpathians and Bieszczady Mountains (a) and samples from all three populations studied (b). Individuals possessing Eastern mtDNA lineage haplotypes are indicated with an asterisk (*), while individuals belonging to Western mtDNA lineage are marked with hash (#)

All *F*_ST_ and *R*_ST_ values are significantly different from zero at *p* < 0.001.

structure analysis identified *K* = 2 genetic groups when complete 113 multilocus brown bear genotypes were analyzed and surprisingly, the Romanian population grouped with Western Carpathians population. About 85% of all individuals studied were assigned to a genetic cluster using *Q* > 0.90 as a threshold. Irrespective of whether the Romanian population was included in the analysis, brown bears from Western Carpathians and Bieszczady Mountains brown bears always formed separate genetic units (Figure 2a,b). Considerable admixture (13.37%) from Western Carpathian brown bear population into the Bieszczady Mountains was found with *K* = 2. On the other hand, the admixture from the Bieszczady Mountains population into Western Carpathians or Romania was very low (2% and 3%, respectively). When usepopinfo was used, with *K* = 3 and migrprior set at 0.05, six individuals in the Bieszczady Mountains were found to be immigrants or F1 backcrosses at *p* < 0.05 to *p* < 0.001: 4 from Western Carpathians (and they all possessed diagnostic for Western Carpathians H1 or H2 mtDNA haplotype; see the next paragraph) and two from Romania with H5 haplotype. These six individuals with the highest inferred ancestry were all males and they were present all over the Bieszczady Mountains population area. geneclass2 and bayesass also found the same four male immigrants from Western Carpathians (*p* < 0.01), however did not detect any immigrants from Romania into the Bieszczady Mountains. All but one pairwise comparison of relatedness between six males with the highest immigrant ancestry revealed they were not related. A single pair showed significant relatedness at the level of cousins. The statistical analysis of pairwise relatedness coefficient between all 17 admixed individuals found in Bieszczady Mountains without usepopinfo revealed four possible parent‐offspring pairs, two full siblings pairs, three half‐siblings pairs, and five cousins pairs, indicating recent effective dispersal and admixture of brown bears from Western Carpathians. Eight other brown bears that possessed H1 or H2 haplotype (diagnostic for WC, see the next paragraph) that were present in Bieszczady Mountains did not show considerable admixture. bayesass found little recent gene flow (about 1%) from the BM and ROM to the WC populations (*m* = 0.0091; 95% CI: 0.0001–0.0394 and *m* = 0.0088; 95% CI: 0.0001–0.0377, respectively). The same was from the ROM to the BM (about 0.5%; *m* = 0.0051; 95% CI: 0.0001–0.0192). The short‐term gene flow from the WC to the BM and from BM to the ROM population was about 2% (*m* = 0.0232; 95% CI: 0.0055–0.0515 and *m* = 0.0264; 95% CI: 0.0007–0.0887, respectively). The largest level of recent gene flow was found from WC to the ROM population (about 25%; *m* = 0.2500; 95% CI: 0.1121–0.3233), but possibly this result was affected by small sample sizes and low differentiation between WC and ROM.

### 3.2 mtDNA analyses

**Figure 3:**
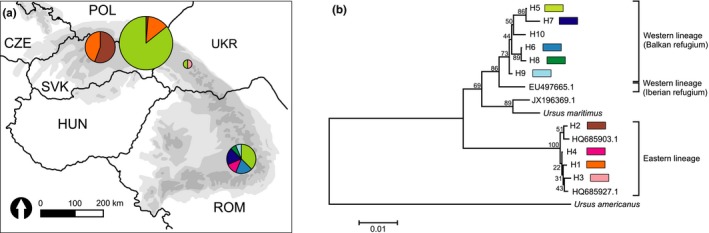
MtDNA haplotype frequencies in studied brown bear populations (a) with respective neighbor‐joining tree based on concatenated mtDNA sequences (b). Size of each diagram is scaled by sample size. Bootstrap support values are given at each node of the tree. The trees have been rooted with sequence of *Ursus americanus*

The analysis of concatenated mtDNA sequences (809–813 bp) yielded nine haplotypes and the tenth haplotype (H10) was found in Greece (Figure 3a,b). The phylogenetic analysis (Figure 3) revealed that all mtDNA haplotypes detected in this study belonged to either the Eastern lineage of brown bears (Taberlet & Bouvet, 1994, e.g., Eurasian lineage; Korsten et al., 2009, Anijalg et al., 2018; clade 3a1, subclade *K*; H1–H4) or the Balkan branch of the Western lineage, for example, clade 1 (H5–H10). The haplotypes from these two distinct clades differed with 28–38 substitutions (Supporting Information Figure S1), which corresponded to the average net divergence *D*a = 3.6% (2.2% for cyt *b* and 5.1% for mtDNA control region). Brown bears from Western Carpathians possessed only haplotypes from the Eastern mtDNA lineage (clade 3a1), while in the Eastern (Slovakia, Poland, and Ukraine) and Romanian Carpathians, both mtDNA lineages (clades 1 and 3a1) were present (Figure 3a). In the brown bear population from the Western Carpathians, we found two mtDNA haplotypes, three in the Bieszczady Mountains, two in Ukraine, and six in Romania (Figure 3a, Supporting Information Table S3). Molecular diversity indices for the brown bear populations studied and for the whole sample at mtDNA are given in Table 3.

**Table 3:** Summary of genetic polymorphism for concatenated mtDNA‐cr and cyt *b* haplotypes in the brown bear from Western Carpathians (WC), Bieszczady Mountains (BM), and Romanian Carpathians (ROM)

Population	*n*_ind_	*N*_h_	*h* (*SD*)	*π* (*SD*)	P	Ti	Tv	Indel	PD (*SD*)
WC									
POL	8	2	0.54 (0.12)	0.001 (0.001)	4	2	0	2	1.07 (0.79)
SVK	17	2	0.53 (0.05)	0.001 (0.001)	5	2	0	3	1.06 (0.74)
POL + SVK	25	2	0.51 (0.04)	0.001 (0.001)	5	2	0	3	1.03 (0.71)
BM									
POL	58	3	0.22 (0.07)	0.009 (0.005)	37	33	1	3	7.13 (3.39)
SVK	18	3	0.45 (0.12)	0.017 (0.009)	37	33	1	3	14.07 (6.62)
POL + SVK	76	3	0.28 (0.06)	0.011 (0.006)	37	33	1	3	8.91 (4.15)
ROM	16	6	0.82 (0.07)	0.013 (0.007)	40	37	1	2	10.33 (4.98)
Total	117	8	0.59 (0.04)	0.019 (0.009)	44	39	1	4	15.26 (6.87)

Note

*n*_ind_: sample size; *N*_h_: number of haplotypes; *h*: haplotype diversity; *π*: nucleotide diversity; *SD*: standard deviation; P: number of segregating sites; Ti: number of transitions; Tv: number of transversions; Indel: number of indels (insertion or deletion); PD: mean number of pairwise differences.

Similar to microsatellite loci, mtDNA pairwise genetic differentiation values between the “transboundary subsamples” from Western Carpathians in Poland and Slovakia were small and not significantly different from zero (*F*_ST_ = 0.000, *p* > 0.05). The same pattern was observed in the Bieszczady Mountains (*F*_ST_ = 0.057, *p* > 0.05). On the other hand, genetic differentiation values for mtDNA between brown bear populations from the Western Carpathians and Bieszczady Mountains were very great (*F*_ST_ = 0.62, *φ*_ST_ = 0.77) and significantly different from zero (*p* < 0.001). The genetic differentiation (*F*_ST_) between population from Romania and other two populations was also great and significant (Table 4). Interestingly, the *φ*_ST_ value (0.02) between Romanian Carpathians and Bieszczady Mountains (POL + SVK) populations did not differ significantly from zero.

**Table 4:** Genetic differentiation in mtDNA (*F*_ST_ above diagonal and *φ*_ST_ below diagonal) between populations of the brown bear from Western Carpathians (WC), Bieszczady Mountains (BM) and Romanian Carpathians (ROM)

*F*_ST_/*φ*_ST_	WC	BM	ROM
WC	–	0.62	0.35
BM	0.77	–	0.29
ROM	0.84	0.02 ns	–

Note

ns: not significantly different from zero. All but one values are statistically significant at *p* < 0.001.

## 4 DISCUSSION

An important finding of this study, that filled a gap in the brown bear phylogeography in Europe, is the dominance of the Eastern mtDNA lineage (clade 3a1, subclade *K*; Anijalg et al., 2018) of the brown bear in the Western Carpathians and the presence of mtDNA haplotypes from both lineages: clade 1b, clade 3a1, as well as genetic admixture in the Eastern and Southern Carpathians (Poland, Slovakia through Ukraine and Romania). The latter result is congruent with the location of contact zone between these brown bear mtDNA lineages postulated by Zachos, Otto, Unici, Lorenzini, and Hartl (2008), and Bray et al. (2013) as well as for other species, such as the bank vole, *Myodes glareolus* (Wójcik, Kawałko, Marková, Searle, & Kotlík, 2010) and the weasel, *Mustela nivalis* (McDevitt et al., 2012). However, we did not detect the Western lineage (clade 1b) in the Western Carpathians within Poland and Slovakia as suggested in Figure 1a by Bray et al. (2013). Since our sample size from the Western Carpathians consisted of 24 individuals, it is possible that the haplotypes of the Western lineage are simply rare in this area. To date, all detected mtDNA sequences reported from the Western Carpathians in Poland and Slovakia belong to clade 3a1. This finding is consistent with Korsten et al. (2009) and Anijalg et al. (2018) who postulated that clade 3a1 experienced a widespread westward expansion after LGM across northern Eurasia and had more western distribution. Our microsatellite and mtDNA data, coupled with a recent genomic study of brown bear in Western and Central Europe (Benazzo et al., 2017), showed a complex scenario for the Western Carpathians. The initial colonization of the Western Carpathians by brown bears possessing mtDNA from subclade *K* of the Eastern lineage could have occurred during the late LGM as shown in Figure 3d of Anijalg et al. (2018). This autochthonous mtDNA could have remained unchanged due to female philopatry (Støen, Zedrosser, Sæbø, & Swenson, 2006; Straka et al., 2012). The colonization process was then followed by population mixing at nuclear loci due to male‐biased dispersal. Indeed, similar allele size ranges at microsatellite loci in bear populations from Western and Eastern Carpathians suggest their genetic affinity, which is in line with the genomic survey (Benazzo et al., 2017) of Apennine, Alpine, Iberian, Balkan, Scandinavian, and Carpathian brown bears. This indicates that there are more complex patterns than previously assumed for brown bears, mtDNA trees alone do not tell the whole story and cannot be tantamount with a given population's evolutionary history and origin.

Straka et al. (2012) in their microsatellite study suggested that the genetic differentiation of Carpathian brown bears in Slovakia and Romania was a result of nearly 100 years of isolation of these geographically close populations. Recent human‐mediated isolation could have helped to maintain clearly visible differentiation at mtDNA. And the genetic drift resulted in genetic differentiation at nuclear loci between Western Carpathian and Bieszczady Mountains (POL and SVK), as well as the Romanian brown bear populations. At present, there must be effective physical barriers obstructing dispersal for both sexes (Fernández, Selva, Yuste, Okarma, & Jakubiec, 2012) and female philopatry (Støen et al., 2006; Straka et al., 2012) alone cannot explain the observed pattern in the studied brown bears that prevent complete mixing. Such barriers between contemporary brown bear populations are likely to function in the form of habitat fragmentation despite the short geographic distance between them. Indeed, Straka et al. (2012) showed a significant genetic divergence between samples from Northern, Central, and Eastern Slovakia that was due to human‐caused fragmentation and isolation. Interestingly, contemporary, asymmetric, male‐biased gene flow from mostly Western Carpathians and less likely from Romania, into the Bieszczady Mountains brown bear population was detected for both mtDNA and microsatellite loci at comparable levels. It is consistent with recent findings of the genetic capture–mark–recapture survey of Paule et al. (2015). The presence of Eastern mtDNA lineage haplotypes in four males identified as immigrants by two approaches based on multilocus microsatellite loci (structure, geneclass2, and bayesass) in the Bieszczady Mountains may indicate recent admixture from the Western Carpathian population that resulted in significant LD among three pairs of microsatellite loci in this population. However, admixture from Western Carpathians soon after World War II may have occurred as other eight brown bears that possessed H1 or H2 haplotypes were present in Bieszczady Mountains, albeit were assigned local ancestry. In addition, the estimate of recent gene flow from the WC to the BM population obtained in bayesass (about 2%) is lower than genetic admixture in the latter population (about 13.37%; structure analysis). This suggests that the observed admixture level cannot be explained by short‐term gene flow between these populations.

Habitat fragmentation is assumed to be a general factor that may increase female philopatry (Henry, Coulon, & Travis, 2016). If we consider that habitat discontinuity and physical obstacles to gene flow might cause genetic differentiation between brown bears even at a relatively small distance in just a few generations of isolation (Straka et al., 2012; Straka, Paule, Štofík, Ionescu, & Adamec, 2011). Then, asymmetric admixture from the Western Carpathians into the Bieszczady Mountains population detected in our study may have also resulted from human‐mediated translocations that recently occurred in the past. In 1982, a female and two cubs plus another female and male (five brown bears in total) that originated from Central Slovakia were released into Eastern Slovakia. Similarly, in 1990, a female and two cubs were translocated from North‐Central Slovakia to the abovementioned area, very close to the Polish border (Štofík, Bural, Paule, & Straka, 2010), possibly resulting in a genetic admixture. Straka et al. (2012) supposed that migration between Western Carpathians and Bieszczady Mountains was excluded. However, Paule et al. (2015) identified two males, which migrated from Western Carpathians to the Bieszczady Mountains via straight‐line (distance about 180–200 km). It is in agreement with the sex‐biased dispersal and asymmetric admixture found in our study.

Brown bears in the Bieszczady Mountains (Poland and Slovakia) after World War II have been a minority (Jakubiec, 2001; Sabadoš & Šimiak, 1981). Thus, detected direction, asymmetry in genetic admixture may indicate that male brown bears migrated to the Bieszczady Mountains from the areas with higher population densities (Western Carpathians and Romania). During population recovery, asymmetric gene flow supports a metapopulation model of genetic connectivity, as shown for black bears (*Ursus americanus*) in the mountainous areas of the western Great Basin, USA (Malaney, Lackey, Beckmann, & Matocq, 2018). All these findings suggest a nonequilibrium, complex scenario of the Carpathian brown bear population recovery after World War II.

Our results suggest that the studied Carpathian brown bear populations, despite being divergent at mtDNA and microsatellites, should not be considered as distinct conservation units (sensu Taberlet, Swenson, Sandegren, & Bjärvall, 1995) as they exhibit genetic affinity at nuclear loci. Significant admixture into the bear population that inhabits Bieszczady Mountains seems to be an effect of natural, male‐biased dispersal as well as human‐mediated translocations that acted in conjunction and caused the observed pattern of genetic diversity in the brown bear populations studied. Secondly, as expected, we showed that the boundary between Poland and Slovakia has no effect on the genetic structure of the brown bear population. Although, successful management of this species will require action plans to be coordinated between the responsible authorities of both countries. Good practices already exist as in the Slovak Tatra National Park (south from Polish Tatra National Park) where culling of brown bears is prohibited. This fact is particularly important as a majority of brown bears in the Western Carpathians reside in Slovakia where they are legally culled, while only a small portion (ca. 15 individuals) live in Poland. On the other hand, most of the Bieszczady Mountains brown bears (ca. 80%) reside in Poland and brown bear hunting has never been permitted in the entire Eastern Carpathians in Slovakia. Thus, for the successful conservation management of brown bears in Poland, Slovakia, and Ukraine, we recommend (a) focus on conservation actions on improving population connectivity and (b) genomic and telemetric studies to be performed or continued, likewise proposed for the Apennine bear population (Benazzo et al., 2017). The most important goal would be to allow brown bear undisturbed dispersal, a factor that until recently, homogenized brown bear populations (Benazzo et al., 2017). To fulfill this demand, connectivity analysis based on actual movement data of bears (Ziółkowska et al., 2016) should be performed, migratory corridors should be identified and incorporated in regional plans of infrastructure and housing development. Fernández et al. (2012), for example, highlighted the need to control unplanned urban sprawl to preserve both brown bear habitats and the connectivity between the Western and Eastern Carpathian populations. To conclude, our genetic survey highlights a pivotal role of transboundary migratory corridors to ensure the long‐lasting existence of brown bear populations in Europe.

## CONFLICT OF INTEREST

The authors declare no competing interests.

## AUTHOR CONTRIBUTIONS

WS, MM, LP, and MR conceived and developed the initial idea; SJ, WS, and MR acquired funding for data collection; WS, LP, JŠ, DK, and A‐TB collected and provided samples; MM and MC conducted laboratory work; MM, WS, and MR analyzed the data; MM, MR, WS, and LP led the writing with contributions from all authors. All authors approved the final version of the manuscript.

## Data Availability

Sequence data have been submitted to the GenBank database under accession numbers: MG254039–MG254048 (for mtDNA cytochrome *b* gene) and MG254049–MG254058 (for mtDNA control region). Sample information and full microsatellite dataset are given in Supporting Information Table S2.
