# Supplementary material for: Genetic differentiation and asymmetric gene flow among Carpathian brown bear (Ursus arctos) populations—Implications for conservation of transboundary populations
Source: Ecol Evol. 2019 Jan 23;9(3):1501–11. doi: 10.1002/ece3.4872 (PMC6374679; doi:10.1002/ece3.4872)
Supplement: Supplementary file 1 [file ECE3-9-1501-s001.docx]

**SUPPORTING INFORMATION**

**Genetic differentiation and asymmetric gene flow among Carpathian brown bear (*Ursus arctos*) populations – implications for conservation of transboundary populations**

Maciej Matosiuk, Wojciech Śmietana, Magdalena Czajkowska, Ladislav Paule, Jozef Štofik, Diana Krajmerová, Andriy-Taras Bashta, Stefan Jakimiuk, Mirosław Ratkiewicz

**Table S1.** Number of alleles per locus (*A*), allele size range (ASR), null allele frequency (*F*_Null_) and allele dropout (ADO) for the total sample of studied brown bear in the Carpathians (Poland, Slovakia and Romania, *n* = 121) and for three populations studied from Western Carpathians (WC), Bieszczady Mountains (BM) and Romanian Carpathians (ROM).

|  | | |  | | | |  | | | *A* | | | ASR | | | *F*_Null_ | | | ADO | | |
| --- | --- | --- | --- | --- | --- | --- | --- | --- | --- | --- | --- | --- | --- | --- | --- | --- | --- | --- | --- | --- | --- |
| Locus | Panel | Dye | | **WC** | **BM** | **ROM** | | **Total** | **WC** | | **BM** | **ROM** | | **Total** | **WC** | | **BM** | **ROM** | | **Total** |  |
| G10J | 1 | NED | | 6 | 5 | 7 | | 7 | 77–99 | | 77–99 | 77–99 | | 77–99 | 0.205 | | 0.045 | 0.197 | | na | 0.010 |
| Mu61 | 1 | FAM | | 4 | 6 | 6 | | 7 | 138–146 | | 138–148 | 138–152 | | 138–152 | 0.000 | | 0.000 | 0.000 | | na | 0.078 |
| Mu09 | 1 | VIC | | 5 | 6 | 7 | | 9 | 184–196 | | 182–200 | 188–202 | | 182–202 | 0.038 | | 0.009 | 0.098 | | na | 0.116 |
| G10M | 1 | FAM | | 5 | 7 | 5 | | 7 | 203–217 | | 203–217 | 209–217 | | 203–217 | 0.078 | | 0.015 | 0.000 | | na | 0.080 |
| Mu59 | 2 | NED | | 11 | 8 | 11 | | 13 | 92–120 | | 92–118 | 94–120 | | 92–120 | 0.000 | | 0.000 | 0.094 | | na | 0.036 |
| G10C | 2 | FAM | | 6 | 7 | 8 | | 8 | 92–110 | | 92–112 | 92–112 | | 92–112 | 0.000 | | 0.108 | 0.093 | | na | 0.029 |
| G10B | 2 | FAM | | 5 | 7 | 7 | | 8 | 135–153 | | 135–153 | 135–153 | | 135–153 | 0.013 | | 0.034 | 0.012 | | na | 0.028 |
| Mu11 | 3 | PET | | 5 | 10 | 6 | | 10 | 83–97 | | 79–101 | 81–95 | | 79–101 | 0.000 | | 0.086 | 0.027 | | na | 0.018 |

na – not applicable.

**Table S2.** List of the 133 samples used in this study: sample ID; country of origin; population; mitochondrial DNA haplotype; sample selection for mtDNA, population genetics and STRUCTURE analyses; sex determination; and corresponding genotype in eight microsatellite loci.

| ID | Country | Population | mtDNA | Haplotype | Pop. Gen. | STRUCTURE | Sex | G10J | | Mu61 | | Mu09 | | G10M | | G10C | | Mu59 | | G10B | | Mu11 | |
| --- | --- | --- | --- | --- | --- | --- | --- | --- | --- | --- | --- | --- | --- | --- | --- | --- | --- | --- | --- | --- | --- | --- | --- |
| 1 | Poland | Western Carpathians | + | H2 | + | **–** | F | 77 | 77 | 146 | 146 | 0 | 0 | 203 | 213 | 92 | 104 | 0 | 0 | 0 | 0 | 83 | 97 |
| 2 | Poland | Western Carpathians | + | H2 | + | **–** | M | 77 | 95 | 146 | 146 | 0 | 0 | 0 | 0 | 92 | 92 | 118 | 118 | 135 | 147 | 83 | 91 |
| 3 | Poland | Western Carpathians | + | H2 | + | **–** | M | 77 | 79 | 144 | 146 | 190 | 192 | 0 | 0 | 92 | 110 | 104 | 118 | 147 | 149 | 93 | 97 |
| 4 | Poland | Western Carpathians | + | H1 | + | + | M | 79 | 95 | 138 | 144 | 190 | 190 | 203 | 211 | 92 | 104 | 92 | 118 | 135 | 147 | 93 | 95 |
| 5 | Poland | Western Carpathians | + | H1 | + | + | F | 79 | 95 | 142 | 144 | 194 | 194 | 211 | 211 | 92 | 104 | 100 | 104 | 147 | 153 | 95 | 95 |
| 6 | Poland | Western Carpathians | + | H2 | + | + | M | 77 | 79 | 146 | 146 | 196 | 196 | 213 | 213 | 92 | 102 | 98 | 116 | 135 | 135 | 83 | 97 |
| 7 | Poland | Western Carpathians | + | H1 | + | + | M | 89 | 95 | 144 | 144 | 196 | 196 | 203 | 211 | 92 | 110 | 104 | 118 | 135 | 147 | 95 | 97 |
| 8 | Slovakia | Western Carpathians | + | H2 | + | + | F | 77 | 95 | 146 | 146 | 184 | 196 | 213 | 217 | 92 | 92 | 118 | 118 | 135 | 147 | 83 | 91 |
| 9 | Slovakia | Western Carpathians | + | H2 | + | + | F | 95 | 99 | 144 | 146 | 194 | 196 | 203 | 209 | 92 | 104 | 110 | 116 | 147 | 153 | 83 | 93 |
| 10 | Slovakia | Western Carpathians | + | H1 | + | + | F | 79 | 99 | 138 | 146 | 194 | 194 | 209 | 211 | 104 | 106 | 110 | 118 | 135 | 147 | 93 | 95 |
| 11 | Slovakia | Western Carpathians | + | H2 | + | + | M | 77 | 99 | 142 | 144 | 194 | 196 | 209 | 209 | 92 | 110 | 98 | 116 | 147 | 147 | 83 | 95 |
| 12 | Slovakia | Western Carpathians | + | H2 | + | + | M | 89 | 99 | 144 | 146 | 194 | 196 | 209 | 209 | 96 | 106 | 100 | 114 | 147 | 147 | 93 | 95 |
| 13 | Slovakia | Western Carpathians | + | H1 | + | + | M | 79 | 89 | 144 | 146 | 190 | 196 | 209 | 211 | 96 | 102 | 118 | 120 | 147 | 149 | 95 | 95 |
| 14 | Slovakia | Western Carpathians | + | H2 | + | + | M | 87 | 95 | 138 | 146 | 190 | 190 | 203 | 209 | 102 | 110 | 112 | 118 | 135 | 147 | 95 | 95 |
| 15 | Slovakia | Western Carpathians | + | H1 | + | + | F | 89 | 99 | 144 | 144 | 194 | 196 | 209 | 211 | 102 | 102 | 110 | 114 | 137 | 147 | 93 | 95 |
| 16 | Slovakia | Western Carpathians | + | H2 | + | + | F | 77 | 95 | 144 | 146 | 192 | 196 | 203 | 213 | 92 | 106 | 110 | 118 | 135 | 135 | 83 | 95 |
| 17 | Slovakia | Western Carpathians | + | H2 | + | **–** | F | 99 | 99 | 138 | 146 | 194 | 196 | 0 | 0 | 92 | 106 | 110 | 118 | 147 | 147 | 93 | 97 |
| 18 | Slovakia | Western Carpathians | + | H2 | + | + | F | 95 | 95 | 146 | 146 | 192 | 196 | 209 | 211 | 92 | 110 | 98 | 116 | 147 | 147 | 83 | 83 |
| 19 | Slovakia | Western Carpathians | + | H2 | + | + | F | 77 | 95 | 144 | 146 | 192 | 196 | 209 | 209 | 92 | 104 | 110 | 118 | 147 | 153 | 95 | 97 |
| 20 | Slovakia | Western Carpathians | + | H1 | + | + | M | 89 | 99 | 144 | 146 | 192 | 194 | 209 | 211 | 92 | 110 | 104 | 110 | 135 | 135 | 95 | 95 |
| 21 | Slovakia | Western Carpathians | + | H1 | + | + | F | 89 | 89 | 144 | 146 | 190 | 196 | 203 | 209 | 92 | 110 | 118 | 118 | 147 | 147 | 83 | 95 |
| 22 | Slovakia | Western Carpathians | + | H1 | + | + | F | 89 | 89 | 138 | 144 | 196 | 196 | 211 | 211 | 92 | 104 | 108 | 116 | 135 | 149 | 93 | 97 |
| 23 | Slovakia | Western Carpathians | + | H1 | + | + | F | 77 | 77 | 142 | 144 | 194 | 196 | 211 | 211 | 104 | 104 | 110 | 118 | 135 | 147 | 95 | 97 |
| 24 | Poland | Bieszczady Mountains | + | H5 | + | + | F | 79 | 99 | 142 | 146 | 196 | 196 | 213 | 213 | 106 | 106 | 98 | 118 | 147 | 147 | 85 | 91 |
| 25 | Poland | Bieszczady Mountains | + | H5 | + | + | M | 79 | 79 | 146 | 148 | 196 | 196 | 213 | 213 | 106 | 110 | 100 | 116 | 147 | 147 | 79 | 85 |
| 26 | Poland | Bieszczady Mountains | + | H1 | + | + | F | 89 | 89 | 144 | 144 | 196 | 196 | 207 | 213 | 110 | 112 | 110 | 110 | 147 | 149 | 91 | 95 |
| 27 | Poland | Bieszczady Mountains | + | H5 | + | + | F | 77 | 79 | 142 | 146 | 196 | 200 | 211 | 211 | 92 | 110 | 110 | 118 | 147 | 151 | 81 | 83 |
| 28 | Poland | Bieszczady Mountains | + | H5 | + | + | M | 79 | 79 | 142 | 148 | 196 | 200 | 213 | 213 | 96 | 110 | 92 | 100 | 147 | 147 | 91 | 93 |
| 29 | Poland | Bieszczady Mountains | + | H5 | + | + | M | 79 | 79 | 148 | 148 | 196 | 196 | 207 | 213 | 92 | 106 | 114 | 118 | 145 | 147 | 85 | 93 |
| 30 | Poland | Bieszczady Mountains | + | H5 | + | + | F | 79 | 79 | 142 | 148 | 184 | 200 | 207 | 213 | 110 | 112 | 92 | 118 | 147 | 149 | 91 | 93 |
| 31 | Poland | Bieszczady Mountains | + | H5 | + | + | F | 77 | 79 | 146 | 146 | 200 | 200 | 211 | 213 | 110 | 110 | 100 | 116 | 149 | 151 | 91 | 101 |
| 32 | Poland | Bieszczady Mountains | + | H5 | + | + | F | 79 | 79 | 142 | 148 | 196 | 196 | 213 | 213 | 92 | 110 | 114 | 116 | 147 | 147 | 85 | 93 |
| 33 | Poland | Bieszczady Mountains | + | H1 | + | + | M | 77 | 89 | 142 | 146 | 200 | 200 | 211 | 213 | 106 | 110 | 118 | 118 | 151 | 151 | 93 | 101 |
| 34 | Poland | Bieszczady Mountains | + | H5 | + | + | M | 79 | 79 | 142 | 142 | 196 | 200 | 207 | 213 | 92 | 110 | 100 | 100 | 147 | 151 | 93 | 97 |
| 35 | Poland | Bieszczady Mountains | + | H1 | + | + | M | 77 | 89 | 142 | 144 | 196 | 200 | 207 | 213 | 110 | 112 | 100 | 110 | 147 | 149 | 91 | 95 |
| 36 | Poland | Bieszczady Mountains | + | H5 | + | + | F | 77 | 79 | 142 | 146 | 196 | 200 | 209 | 211 | 92 | 112 | 110 | 118 | 147 | 147 | 83 | 95 |
| 37 | Poland | Bieszczady Mountains | + | H5 | + | + | M | 77 | 79 | 142 | 148 | 196 | 200 | 207 | 213 | 110 | 112 | 100 | 118 | 149 | 149 | 91 | 93 |
| 38 | Poland | Bieszczady Mountains | + | H5 | + | + | F | 79 | 99 | 144 | 148 | 196 | 200 | 207 | 209 | 96 | 110 | 100 | 100 | 135 | 147 | 93 | 101 |
| 39 | Poland | Bieszczady Mountains | + | H1 | + | + | M | 79 | 79 | 138 | 144 | 192 | 196 | 207 | 211 | 92 | 110 | 100 | 110 | 135 | 145 | 93 | 97 |
| 40 | Poland | Bieszczady Mountains | + | H5 | + | + | M | 79 | 79 | 142 | 144 | 196 | 196 | 209 | 215 | 106 | 110 | 110 | 114 | 147 | 151 | 83 | 101 |
| 41 | Poland | Bieszczady Mountains | + | H5 | + | **–** | F | 79 | 99 | 146 | 148 | 196 | 200 | 0 | 0 | 110 | 110 | 100 | 118 | 147 | 151 | 83 | 91 |
| 42 | Poland | Bieszczady Mountains | + | H1 | + | + | M | 89 | 99 | 142 | 144 | 194 | 196 | 209 | 211 | 92 | 110 | 98 | 118 | 147 | 149 | 87 | 95 |
| 43 | Poland | Bieszczady Mountains | + | H5 | + | + | M | 79 | 79 | 142 | 148 | 196 | 200 | 207 | 207 | 96 | 112 | 100 | 118 | 147 | 151 | 91 | 93 |
| 44 | Poland | Bieszczady Mountains | + | H5 | + | + | F | 79 | 79 | 144 | 148 | 196 | 200 | 207 | 211 | 96 | 110 | 92 | 100 | 135 | 147 | 93 | 93 |
| 45 | Poland | Bieszczady Mountains | + | H5 | + | + | F | 79 | 79 | 142 | 148 | 196 | 196 | 207 | 213 | 92 | 96 | 100 | 114 | 145 | 147 | 91 | 93 |
| 46 | Poland | Bieszczady Mountains | + | H5 | + | + | F | 77 | 99 | 144 | 146 | 182 | 200 | 213 | 213 | 110 | 112 | 98 | 110 | 149 | 151 | 85 | 95 |
| 47 | Poland | Bieszczady Mountains | + | H5 | + | + | M | 79 | 79 | 138 | 148 | 196 | 200 | 207 | 213 | 92 | 112 | 100 | 118 | 145 | 151 | 93 | 93 |
| 48 | Poland | Bieszczady Mountains | + | H5 | + | + | M | 79 | 79 | 142 | 148 | 196 | 196 | 209 | 213 | 92 | 110 | 100 | 118 | 147 | 147 | 83 | 91 |
| 49 | Poland | Bieszczady Mountains | + | H5 | + | + | F | 79 | 79 | 144 | 148 | 196 | 196 | 207 | 213 | 92 | 106 | 100 | 100 | 145 | 147 | 85 | 97 |
| 50 | Poland | Bieszczady Mountains | + | H5 | + | + | F | 79 | 99 | 146 | 146 | 182 | 200 | 213 | 213 | 106 | 110 | 98 | 100 | 147 | 151 | 85 | 101 |
| 51 | Poland | Bieszczady Mountains | + | H5 | + | + | F | 79 | 79 | 142 | 144 | 192 | 200 | 207 | 207 | 92 | 112 | 110 | 118 | 135 | 135 | 93 | 93 |
| 52 | Poland | Bieszczady Mountains | + | H5 | + | + | M | 79 | 89 | 138 | 144 | 196 | 196 | 207 | 213 | 110 | 112 | 100 | 100 | 145 | 149 | 91 | 93 |
| 53 | Poland | Bieszczady Mountains | + | H5 | + | + | M | 77 | 79 | 144 | 146 | 196 | 200 | 207 | 211 | 110 | 110 | 100 | 110 | 149 | 149 | 91 | 97 |
| 54 | Poland | Bieszczady Mountains | + | H5 | + | + | F | 79 | 79 | 146 | 148 | 196 | 200 | 211 | 213 | 92 | 96 | 92 | 118 | 147 | 147 | 93 | 95 |
| 55 | Poland | Bieszczady Mountains | + | H5 | + | + | M | 79 | 89 | 138 | 142 | 196 | 200 | 211 | 213 | 110 | 112 | 110 | 110 | 145 | 147 | 91 | 97 |
| 56 | Poland | Bieszczady Mountains | + | H5 | + | + | M | 77 | 99 | 142 | 146 | 200 | 200 | 213 | 213 | 106 | 110 | 100 | 100 | 147 | 149 | 85 | 91 |
| 57 | Poland | Bieszczady Mountains | + | H1 | + | + | M | 79 | 99 | 146 | 146 | 194 | 196 | 209 | 211 | 92 | 110 | 110 | 110 | 147 | 153 | 93 | 95 |
| 58 | Poland | Bieszczady Mountains | + | H5 | + | + | F | 79 | 79 | 142 | 144 | 196 | 200 | 207 | 207 | 92 | 96 | 92 | 100 | 147 | 151 | 93 | 97 |
| 59 | Poland | Bieszczady Mountains | + | H5 | + | + | M | 77 | 95 | 144 | 146 | 192 | 200 | 203 | 213 | 110 | 110 | 100 | 104 | 151 | 151 | 101 | 101 |
| 60 | Poland | Bieszczady Mountains | + | H5 | + | + | F | 77 | 79 | 144 | 146 | 192 | 196 | 211 | 213 | 106 | 110 | 98 | 100 | 147 | 147 | 85 | 95 |
| 61 | Poland | Bieszczady Mountains | + | H5 | + | + | F | 79 | 99 | 144 | 144 | 196 | 196 | 207 | 207 | 96 | 106 | 100 | 110 | 145 | 147 | 93 | 101 |
| 62 | Poland | Bieszczady Mountains | + | H5 | + | + | F | 79 | 79 | 146 | 148 | 196 | 196 | 211 | 213 | 96 | 106 | 118 | 118 | 147 | 147 | 79 | 93 |
| 63 | Poland | Bieszczady Mountains | + | H5 | + | + | F | 79 | 79 | 138 | 144 | 196 | 200 | 209 | 211 | 96 | 110 | 92 | 110 | 135 | 145 | 93 | 101 |
| 64 | Poland | Bieszczady Mountains | + | H5 | + | + | F | 79 | 89 | 144 | 144 | 184 | 196 | 207 | 207 | 110 | 112 | 92 | 110 | 135 | 149 | 91 | 97 |
| 65 | Poland | Bieszczady Mountains | + | H5 | + | **–** | F | 77 | 79 | 142 | 142 | 196 | 196 | 0 | 0 | 92 | 96 | 100 | 110 | 147 | 147 | 91 | 95 |
| 66 | Poland | Bieszczady Mountains | + | H5 | + | + | M | 77 | 79 | 142 | 146 | 182 | 200 | 211 | 213 | 106 | 110 | 100 | 118 | 147 | 151 | 85 | 101 |
| 67 | Poland | Bieszczady Mountains | + | H5 | + | + | F | 79 | 89 | 142 | 148 | 196 | 196 | 209 | 213 | 106 | 110 | 110 | 118 | 147 | 153 | 95 | 97 |
| 68 | Poland | Bieszczady Mountains | + | H5 | + | + | F | 79 | 79 | 142 | 144 | 192 | 200 | 207 | 211 | 92 | 112 | 100 | 118 | 135 | 151 | 93 | 97 |
| 69 | Poland | Bieszczady Mountains | + | H5 | + | + | F | 79 | 79 | 148 | 148 | 196 | 200 | 207 | 207 | 106 | 110 | 92 | 114 | 135 | 147 | 93 | 93 |
| 70 | Poland | Bieszczady Mountains | + | H5 | + | + | M | 79 | 79 | 138 | 148 | 192 | 200 | 207 | 211 | 110 | 110 | 92 | 100 | 135 | 147 | 93 | 93 |
| 71 | Poland | Bieszczady Mountains | + | H5 | + | + | F | 79 | 99 | 146 | 146 | 196 | 200 | 211 | 213 | 106 | 110 | 98 | 118 | 151 | 151 | 87 | 91 |
| 72 | Poland | Bieszczady Mountains | + | H5 | + | + | F | 77 | 79 | 142 | 146 | 196 | 196 | 213 | 213 | 110 | 110 | 100 | 118 | 149 | 151 | 91 | 95 |
| 73 | Poland | Bieszczady Mountains | + | H5 | + | + | M | 79 | 79 | 140 | 148 | 182 | 200 | 209 | 213 | 92 | 110 | 100 | 104 | 135 | 149 | 83 | 91 |
| 74 | Poland | Bieszczady Mountains | **–** |  | + | + | F | 79 | 89 | 142 | 146 | 196 | 200 | 213 | 215 | 106 | 110 | 100 | 110 | 151 | 151 | 91 | 101 |
| 75 | Poland | Bieszczady Mountains | **–** |  | + | + | F | 77 | 79 | 142 | 146 | 196 | 200 | 211 | 213 | 110 | 112 | 100 | 100 | 147 | 149 | 95 | 101 |
| 76 | Poland | Bieszczady Mountains | **–** |  | + | + | M | 77 | 79 | 142 | 146 | 196 | 196 | 211 | 211 | 110 | 112 | 92 | 100 | 147 | 149 | 93 | 95 |
| 77 | Poland | Bieszczady Mountains | **–** |  | + | + | F | 79 | 79 | 142 | 148 | 196 | 196 | 213 | 213 | 110 | 110 | 100 | 114 | 147 | 147 | 93 | 93 |
| 78 | Poland | Bieszczady Mountains | + | H5 | + | + | M | 77 | 79 | 142 | 148 | 196 | 196 | 211 | 213 | 110 | 110 | 92 | 116 | 147 | 151 | 83 | 101 |
| 79 | Poland | Bieszczady Mountains | + | H2 | + | + | M | 89 | 89 | 144 | 146 | 194 | 196 | 203 | 213 | 102 | 104 | 98 | 98 | 147 | 149 | 95 | 95 |
| 80 | Poland | Bieszczady Mountains | + | H5 | + | + | F | 79 | 79 | 146 | 148 | 200 | 200 | 209 | 213 | 92 | 110 | 100 | 118 | 147 | 147 | 93 | 95 |
| 81 | Poland | Bieszczady Mountains | + | H5 | + | + | F | 79 | 99 | 144 | 144 | 196 | 196 | 207 | 209 | 96 | 106 | 100 | 110 | 145 | 147 | 93 | 101 |
| 82 | Poland | Bieszczady Mountains | **–** |  | + | + | M | 79 | 79 | 144 | 148 | 192 | 196 | 207 | 209 | 96 | 110 | 92 | 110 | 135 | 145 | 93 | 101 |
| 83 | Poland | Bieszczady Mountains | + | H5 | + | + | F | 79 | 79 | 142 | 146 | 196 | 200 | 211 | 211 | 110 | 110 | 100 | 116 | 149 | 149 | 91 | 93 |
| 84 | Poland | Bieszczady Mountains | + | H5 | + | + | M | 77 | 89 | 144 | 146 | 184 | 184 | 207 | 217 | 92 | 110 | 110 | 118 | 135 | 135 | 83 | 97 |
| 85 | Poland | Bieszczady Mountains | **–** |  | + | + | F | 79 | 79 | 142 | 144 | 196 | 200 | 213 | 213 | 96 | 106 | 100 | 100 | 145 | 147 | 85 | 93 |
| 86 | Poland | Bieszczady Mountains | + | H5 | + | + | F | 79 | 99 | 146 | 148 | 196 | 196 | 207 | 209 | 96 | 106 | 114 | 116 | 147 | 153 | 93 | 97 |
| 87 | Poland | Bieszczady Mountains | **–** |  | + | + | M | 79 | 99 | 142 | 146 | 196 | 196 | 211 | 211 | 106 | 112 | 92 | 116 | 147 | 151 | 87 | 93 |
| 88 | Poland | Bieszczady Mountains | **–** |  | + | + | M | 79 | 79 | 142 | 146 | 196 | 196 | 211 | 213 | 106 | 112 | 92 | 116 | 147 | 151 | 87 | 93 |
| 89 | Poland | Bieszczady Mountains | + | H5 | + | + | M | 79 | 79 | 148 | 148 | 196 | 196 | 211 | 213 | 96 | 106 | 92 | 100 | 135 | 147 | 85 | 93 |
| 90 | Slovakia | Bieszczady Mountains | **–** |  | + | + | F | 79 | 89 | 142 | 144 | 184 | 196 | 213 | 213 | 112 | 112 | 92 | 100 | 147 | 147 | 93 | 95 |
| 91 | Slovakia | Bieszczady Mountains | + | H5 | + | + | M | 77 | 79 | 142 | 146 | 200 | 200 | 207 | 213 | 110 | 110 | 100 | 118 | 147 | 149 | 91 | 91 |
| 92 | Slovakia | Bieszczady Mountains | + | H2 | + | + | M | 77 | 95 | 142 | 142 | 196 | 196 | 211 | 215 | 92 | 110 | 114 | 118 | 147 | 147 | 83 | 101 |
| 93 | Slovakia | Bieszczady Mountains | + | H5 | + | + | M | 77 | 79 | 142 | 148 | 200 | 200 | 207 | 213 | 112 | 112 | 100 | 118 | 147 | 149 | 91 | 93 |
| 94 | Slovakia | Bieszczady Mountains | + | H5 | + | + | F | 79 | 89 | 142 | 144 | 184 | 200 | 207 | 207 | 92 | 112 | 100 | 110 | 147 | 149 | 93 | 95 |
| 95 | Slovakia | Bieszczady Mountains | + | H5 | + | + | M | 79 | 89 | 144 | 148 | 184 | 196 | 207 | 213 | 110 | 110 | 92 | 110 | 147 | 149 | 91 | 95 |
| 96 | Slovakia | Bieszczady Mountains | + | H1 | + | + | M | 89 | 89 | 142 | 148 | 184 | 196 | 211 | 213 | 106 | 110 | 100 | 110 | 147 | 147 | 87 | 91 |
| 97 | Slovakia | Bieszczady Mountains | + | H1 | + | + | M | 77 | 89 | 142 | 146 | 196 | 200 | 211 | 211 | 106 | 110 | 92 | 100 | 151 | 151 | 87 | 101 |
| 98 | Slovakia | Bieszczady Mountains | + | H1 | + | + | M | 77 | 89 | 146 | 146 | 192 | 200 | 211 | 213 | 110 | 110 | 100 | 116 | 137 | 151 | 85 | 101 |
| 99 | Slovakia | Bieszczady Mountains | + | H5 | + | + | F | 79 | 89 | 142 | 146 | 200 | 200 | 213 | 213 | 110 | 110 | 100 | 116 | 147 | 151 | 91 | 95 |
| 100 | Slovakia | Bieszczady Mountains | + | H5 | + | + | M | 77 | 79 | 142 | 142 | 196 | 200 | 207 | 213 | 110 | 110 | 100 | 118 | 147 | 147 | 91 | 95 |
| 101 | Slovakia | Bieszczady Mountains | + | H5 | + | + | M | 77 | 79 | 142 | 148 | 184 | 196 | 207 | 213 | 110 | 110 | 100 | 110 | 147 | 149 | 91 | 95 |
| 102 | Slovakia | Bieszczady Mountains | **–** |  | + | + | F | 79 | 89 | 146 | 148 | 192 | 196 | 211 | 211 | 106 | 110 | 100 | 100 | 147 | 151 | 87 | 93 |
| 103 | Slovakia | Bieszczady Mountains | **–** |  | + | + | F | 77 | 79 | 138 | 146 | 196 | 200 | 209 | 211 | 92 | 110 | 92 | 116 | 135 | 149 | 93 | 101 |
| 104 | Slovakia | Bieszczady Mountains | **–** |  | + | + | M | 79 | 89 | 144 | 146 | 196 | 200 | 207 | 213 | 110 | 112 | 110 | 116 | 149 | 151 | 87 | 95 |
| 105 | Slovakia | Bieszczady Mountains | **–** |  | + | + | M | 79 | 79 | 138 | 146 | 196 | 200 | 209 | 211 | 110 | 112 | 92 | 92 | 145 | 149 | 91 | 93 |
| 106 | Slovakia | Bieszczady Mountains | **–** |  | + | + | M | 79 | 79 | 142 | 146 | 196 | 196 | 211 | 213 | 96 | 110 | 110 | 116 | 147 | 149 | 83 | 97 |
| 107 | Romania | Romanian Carpathians | + | H5 | + | + | F | 91 | 95 | 144 | 148 | 196 | 200 | 209 | 209 | 92 | 110 | 104 | 110 | 135 | 149 | 93 | 93 |
| 108 | Romania | Romanian Carpathians | + | H5 | + | + | F | 79 | 79 | 146 | 148 | 194 | 202 | 211 | 217 | 102 | 104 | 98 | 104 | 135 | 147 | 93 | 95 |
| 109 | Romania | Romanian Carpathians | + | H5 | + | + | F | 79 | 87 | 144 | 146 | 192 | 192 | 209 | 211 | 92 | 92 | 110 | 114 | 135 | 135 | 95 | 95 |
| 110 | Romania | Romanian Carpathians | + | H6 | + | + | F | 99 | 99 | 144 | 148 | 188 | 192 | 211 | 213 | 100 | 104 | 116 | 118 | 137 | 149 | 81 | 91 |
| 111 | Romania | Romanian Carpathians | + | H6 | + | + | F | 95 | 95 | 144 | 146 | 196 | 200 | 209 | 211 | 102 | 102 | 114 | 116 | 147 | 147 | 87 | 93 |
| 112 | Romania | Romanian Carpathians | + | H5 | + | **–** | F | 77 | 79 | 146 | 146 | 0 | 0 | 0 | 0 | 102 | 102 | 118 | 120 | 137 | 147 | 83 | 95 |
| 113 | Romania | Romanian Carpathians | + | H4 | + | + | M | 79 | 89 | 148 | 152 | 192 | 196 | 209 | 211 | 102 | 102 | 118 | 118 | 147 | 149 | 81 | 93 |
| 114 | Romania | Romanian Carpathians | + | H7 | + | **–** | F | 89 | 95 | 146 | 146 | 192 | 194 | 211 | 213 | 102 | 104 | 0 | 0 | 137 | 141 | 93 | 95 |
| 115 | Romania | Romanian Carpathians | + | H7 | + | + | F | 77 | 79 | 138 | 144 | 192 | 192 | 211 | 215 | 102 | 110 | 94 | 120 | 147 | 153 | 91 | 93 |
| 116 | Romania | Romanian Carpathians | + | H7 | + | + | F | 79 | 79 | 146 | 148 | 192 | 194 | 211 | 213 | 104 | 110 | 116 | 120 | 147 | 147 | 95 | 95 |
| 117 | Romania | Romanian Carpathians | + | H8 | + | + | F | 79 | 95 | 142 | 144 | 200 | 202 | 209 | 209 | 106 | 112 | 118 | 118 | 137 | 149 | 93 | 95 |
| 118 | Romania | Romanian Carpathians | + | H4 | + | + | M | 79 | 79 | 144 | 144 | 196 | 196 | 211 | 211 | 102 | 102 | 108 | 114 | 135 | 137 | 93 | 95 |
| 119 | Romania | Romanian Carpathians | + | H9 | + | + | M | 79 | 91 | 146 | 148 | 192 | 194 | 213 | 215 | 102 | 110 | 108 | 118 | 141 | 145 | 83 | 83 |
| 120 | Romania | Romanian Carpathians | + | H5 | + | + | M | 91 | 91 | 146 | 148 | 192 | 192 | 209 | 211 | 96 | 106 | 100 | 116 | 135 | 137 | 81 | 95 |
| 121 | Romania | Romanian Carpathians | + | H6 | + | + | M | 89 | 89 | 138 | 144 | 190 | 196 | 209 | 209 | 104 | 110 | 96 | 116 | 147 | 147 | 87 | 93 |
| 122 | Poland | Western Carpathians | + | H2 | **–** | **–** | F | 77 | 95 | 144 | 146 | 190 | 194 | 0 | 0 | 90 | 106 | 100 | 100 | 147 | 147 | 95 | 95 |
| 123 | Slovakia | Western Carpathians | + | H1 | **–** | **–** | **–** | 0 | 0 | 0 | 0 | 0 | 0 | 0 | 0 | 0 | 0 | 0 | 0 | 0 | 0 | 0 | 0 |
| 124 | Slovakia | Bieszczady Mountains | + | H5 | **–** | **–** | **–** | 0 | 0 | 0 | 0 | 0 | 0 | 0 | 0 | 0 | 0 | 0 | 0 | 0 | 0 | 0 | 0 |
| 125 | Slovakia | Bieszczady Mountains | + | H5 | **–** | **–** | **–** | 0 | 0 | 0 | 0 | 0 | 0 | 0 | 0 | 0 | 0 | 0 | 0 | 0 | 0 | 0 | 0 |
| 126 | Slovakia | Bieszczady Mountains | + | H1 | **–** | **–** | **–** | 0 | 0 | 0 | 0 | 0 | 0 | 0 | 0 | 0 | 0 | 0 | 0 | 0 | 0 | 0 | 0 |
| 127 | Slovakia | Bieszczady Mountains | + | H5 | **–** | **–** | **–** | 0 | 0 | 0 | 0 | 0 | 0 | 0 | 0 | 0 | 0 | 0 | 0 | 0 | 0 | 0 | 0 |
| 128 | Slovakia | Bieszczady Mountains | + | H5 | **–** | **–** | **–** | 0 | 0 | 0 | 0 | 0 | 0 | 0 | 0 | 0 | 0 | 0 | 0 | 0 | 0 | 0 | 0 |
| 129 | Slovakia | Bieszczady Mountains | + | H5 | **–** | **–** | **–** | 0 | 0 | 0 | 0 | 0 | 0 | 0 | 0 | 0 | 0 | 0 | 0 | 0 | 0 | 0 | 0 |
| 130 | Ukraine | Ukrainian Carpathians | + | H5 | **–** | **–** | **–** | 0 | 0 | 0 | 0 | 0 | 0 | 0 | 0 | 0 | 0 | 0 | 0 | 0 | 0 | 0 | 0 |
| 131 | Ukraine | Ukrainian Carpathians | + | H3 | **–** | **–** | **–** | 0 | 0 | 0 | 0 | 0 | 0 | 0 | 0 | 0 | 0 | 0 | 0 | 0 | 0 | 0 | 0 |
| 132 | Greece | Greece | + | H10 | **–** | **–** | **–** | 0 | 0 | 0 | 0 | 0 | 0 | 0 | 0 | 0 | 0 | 0 | 0 | 0 | 0 | 0 | 0 |
| 133 | Greece | Greece | + | H10 | **–** | **–** | **–** | 0 | 0 | 0 | 0 | 0 | 0 | 0 | 0 | 0 | 0 | 0 | 0 | 0 | 0 | 0 | 0 |

**Table S3.** MtDNA haplotype frequencies in brown bear (*Ursus arctos*) populations from Western Carpathians (WC), Bieszczady Mountains (BM), Ukraine (UKR) and Romanian Carpathians (ROM) based on concatenated control region and cytochrome *b* sequences.

| Population | | H1 | H2 | H3 | H4 | H5 | H6 | H7 | H8 | H9 |
| --- | --- | --- | --- | --- | --- | --- | --- | --- | --- | --- |
| **WC** | POL | 0.375 | 0.625 | – | – | – | – | – | – | – |
|  | SVK | 0.471 | 0.529 | – | – | – | – | – | – | – |
|  | POL+SVK | 0.440 | 0.560 | – | – | – | – | – | – | – |
| **BM** | POL | 0.103 | 0.017 | – | – | 0.879 | – | – | – | – |
|  | SVK | 0.222 | 0.056 | – | – | 0.722 | – | – | – | – |
|  | POL+SVK | 0.132 | 0.026 | – | – | 0.842 | – | – | – | – |
| **UKR** |  | – | – | 0.500 | – | 0.500 | – | – | – | – |
| **ROM** |  | – | – | – | 0.125 | 0.375 | 0.188 | 0.188 | 0.063 | 0.063 |
| **Total** | | 0.176 | 0.134 | 0.008 | 0.017 | 0.597 | 0.025 | 0.025 | 0.008 | 0.008 |

**
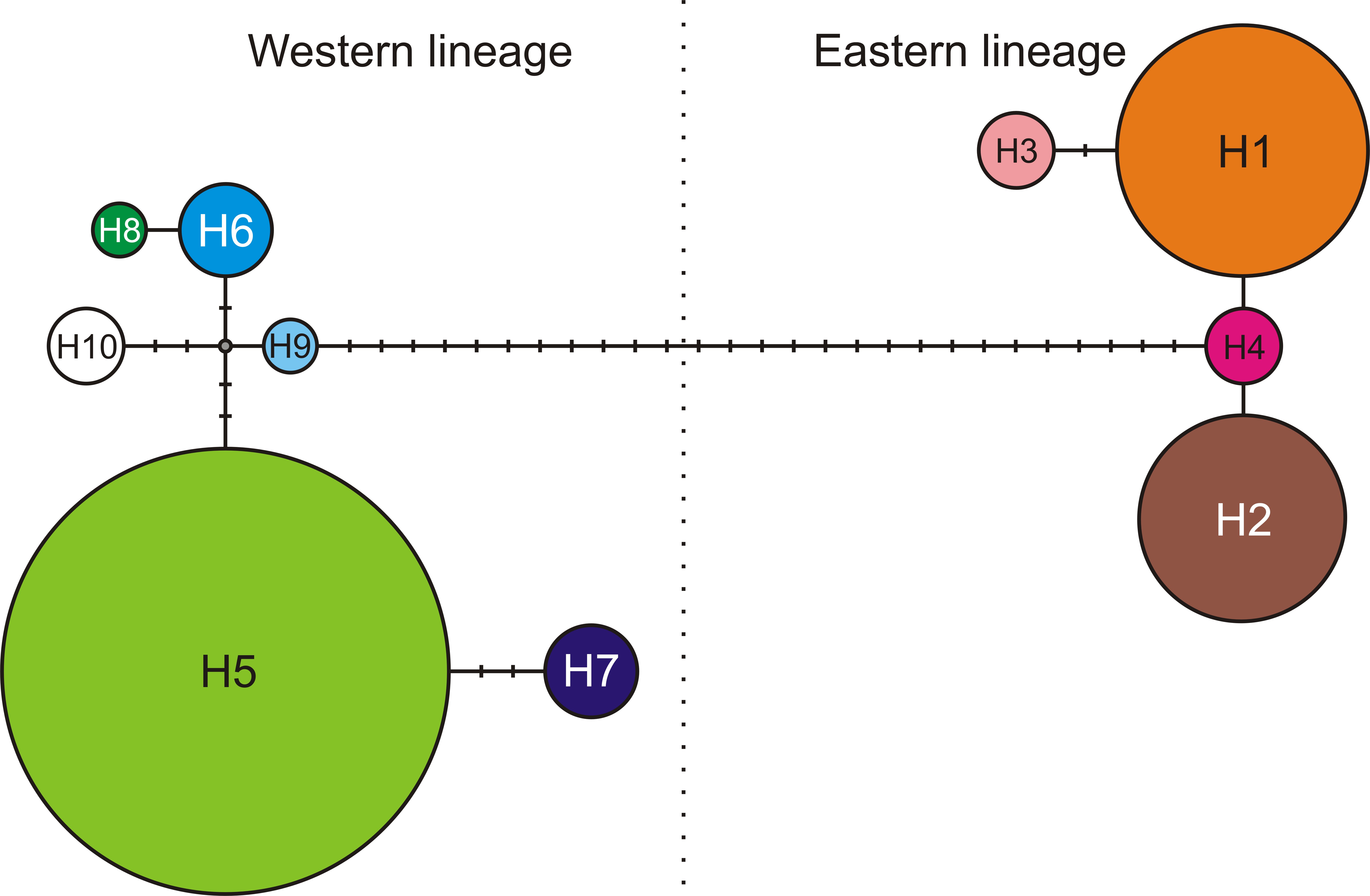
**

**Figure S1.** Median-joining network based on concatenated mtDNA control region and cyt *b* brown bear sequences. Each circle represents a single haplotype and is scaled by its frequency in the whole sample.
